# Heart Rate Variability Nomogram Predicts Atrial Fibrillation in Patients with Moderate to High Burden of Premature Ventricular Complexes

**DOI:** 10.3390/medicina62020243

**Published:** 2026-01-23

**Authors:** Koray Kalenderoglu, Mert Ilker Hayiroglu, Tufan Cinar, Faysal Saylik, Gokcem Ayan Bayraktar, Melih Oz, Miray Ozer Oz, Kadir Gurkan, Tolga Aksu

**Affiliations:** 1 Department of Cardiology, Health Sciences University, Dr. Siyami Ersek Cardiovascular and Thoracic Surgery Hospital, 34668 Istanbul, Turkey; mertilkerh@gmail.com (M.I.H.); mirayozer97@gmail.com (M.O.O.); kadirgrkn@gmail.com (K.G.); 2 Department of Medicine, University of Maryland Midtown Campus, Baltimore, MD 21201, USA; drtufancinar@gmail.com; 3 Department of Cardiology, Van Education and Research Hospital, 65300 Van, Turkey; faysalsaylik@gmail.com; 4 Department of Cardiology, Umraniye Training and Research Hospital, 34760 Istanbul, Turkey; ayangokcem@gmail.com (G.A.B.); melihozz3334@gmail.com (M.O.); 5 Department of Cardiology, Istanbul Aydin University, Medicalpark Florya Hospital, 34295 Istanbul, Turkey

**Keywords:** heart rate variability, premature ventricular complex, atrial fibrillation

## Abstract

*Background and Objectives:* There is a well-established correlation between premature ventricular contractions (PVCs) and atrial fibrillation (AF), with a higher burden of PVCs increasing the likelihood of new-onset AF. This study aims to investigate the impact of heart rate variability (HRV) on the onset of AF in patients with moderate to high burdens of PVCs, as observed through 24 h ambulatory electrocardiogram (ECG) analysis. *Materials and Methods:* Our study was a retrospective analysis involving 187 patients at a single tertiary center. We analyzed PVC counts from 24 h ECG recordings, categorizing the patients into groups based on whether they developed AF or not. Additionally, we developed a nomogram to estimate the risk of AF development in these patients. *Results:* A new-onset AF was detected in 16% of the cohort. Analysis of 24 h ambulatory ECG data revealed statistically significant increases in the SDNN index, RMSSD, PNN50, total power (TP), and low-frequency (LF) values in AF patients. To estimate the risk of AF, a risk prediction nomogram was created using high-frequency (HF), LF, SDNN index, and PNN50. Among these variables, PNN50 was identified as the strongest predictor in the multivariable model. Additionally, a decision curve analysis demonstrated that the nomogram offers a net clinical benefit for detecting AF in patients when the baseline threshold risk exceeds 15%. *Conclusions:* Our study found that among patients with AF who had a moderate to high burden of PVCs using 24 h ambulatory ECGs, several HRV parameters were elevated. This increased autonomic instability may play a role in the development and persistence of AF episodes.

## 1. Introduction

Premature ventricular complex (PVC) is a common cardiac arrhythmia that affects about 1% of the general population and is associated with increased cardiovascular morbidity and mortality [1,2]. Symptoms of PVCs can range from palpitations, chest tightness, and the sensation of skipped beats to more severe issues like presyncope and syncope. In some cases, PVCs may lead to serious conditions such as PVC-induced cardiomyopathy or further ventricular tachyarrhythmias like ventricular tachycardia (VT) and fibrillation, beyond the simple symptoms experienced [3,4]. While the exact underlying mechanisms of PVCs are not fully understood, potential factors include increased automaticity, triggered activity, and re-entry phenomena. Additionally, the incidence of PVCs can be influenced by factors such as coronary heart disease, arterial hypertension (HT), smoking, lack of physical activity, older age, and greater height [5].

Atrial fibrillation (AF) is a widespread arrhythmia that profoundly affects morbidity and mortality, comparable to the risks associated with PVCs [6,7]. Identifying patients at risk for AF is imperative, as this condition can lead to severe and life-threatening complications, including thromboembolism, stroke, congestive heart failure, and increased mortality [8]. The increased probability of AF development in patients with PVCs is a recent subject of discourse, with supporting research available in the literature [9,10,11]. Diverse perspectives exist concerning its pathophysiology. It is thought that AF increases as a consequence of cardiomyopathy developing in patients with a high burden of premature ventricular contractions (PVCs), that AF arises in these patients due to pro-arrhythmic retrograde activation of the atria, or that excessive vagal or sympathetic activity induces AF by decreasing atrial refractoriness and triggering premature atrial contractions [12,13,14,15]. This underscores the importance of vigilant monitoring and timely intervention for patients exhibiting these arrhythmias.

AF and PVCs are profoundly influenced by the autonomic nervous system, encompassing both the sympathetic and parasympathetic branches [16,17]. This autonomic nerve system, alongside the endocrine and immune frameworks, plays a critical role in maintaining homeostasis by regulating physiological functions in response to internal and external challenges [18]. Heart rate variability (HRV) serves as a valuable, noninvasive metric that can be easily obtained through a 24 h ambulatory electrocardiogram (ECG), facilitating the diagnosis of autonomic dysfunction [19]. Diminished HRV levels indicate insufficient adaptation due to autonomic nerve system impairment, while elevated values signify robust adaptability and optimal functioning of autonomic mechanisms [20]. Despite the growing body of research, there remains a conspicuous gap in understanding the relationship between PVCs, AF, and HRV. This study aims to bridge that gap by rigorously examining the influence of HRV on the onset of AF in patients with a moderate to high burden of PVCs, utilizing comprehensive 24 h ambulatory ECG analysis.

## 2. Materials and Methods

### 2.1. Study Population and Design

Our study was designed as a retrospective analysis conducted between May 2016 and July 2018 and involving 187 patients at a single tertiary center. The participants included symptomatic patients who had recorded isolated PVCs during 24 h ambulatory ECG monitoring and were followed by our hospital’s arrhythmia clinic with 24 h ambulatory ECGs at six-month intervals for a minimum of five years following the diagnosis. Symptom status was defined by the presence of palpitations, dyspnea, dizziness, or presyncope temporally associated with PVCs. The study comprised patients without a prior diagnosis of AF. The follow-up data were collected until November 2023. Exclusion criteria included patients younger than 18 years, those without regular ECGs at six-month intervals and 24 h ambulatory ECG recordings, asymptomatic patients, those who did not receive a transthoracic echocardiography (TTE) within three months before the first and last 24 h ECG records, patients with pacemakers, and those being treated with Vaughan Williams class 1 or class 3 antiarrhythmic medications prior to 24 h ambulatory ECG assessments. Data collection encompassed ECG results, 24 h ambulatory ECG recordings, demographic information, TTE measurements, laboratory studies, and medication details, all sourced from the hospital records. PVC counts from the 24 h ECG recordings categorized the patients into three groups: those with fewer than 1000 PVCs were considered as having lower PVC burden; those with between 1000 and 10,000 PVCs were classified as having moderate PVC burden; and those with 10,000 or more PVCs were labeled as having higher PVC burden [11]. New-onset AF was characterized as the initial or first identifiable occurrence of persistent or paroxysmal AF. The AF detected in the study’s patients included both those diagnosed after the emergence of new symptoms and asymptomatic patients in whom AF was discovered during follow-up using ECG or 24 h ambulatory ECG monitoring.

All patients underwent the standard TTE using a 2.5–3.5 MHz transducer (the Philips EPIQ 7C System from Philips Healthcare, Andover, MA, USA), and all studies were conducted by skilled echocardiographers. The transmission and analysis of 24 h ambulatory ECG records were supported by multichannel electronic data recording systems. Data acquisition was performed under standardized ambulatory conditions, and patients were instructed to maintain their usual daily activities while avoiding excessive physical exertion during monitoring. The ECG data were transmitted from the DMS300-4A Holter ECG recorder (DM software Inc., Stateline, NV, USA) to a specialized computer equipped with the Cardio-Scan Premier 12 software version 12.4.0051a (Core Diagnostics Pty. Ltd., Bondi Junction, Australia). The methods utilized in this study adhered to the standards set forth in the Declaration of Helsinki and its amendments, and the study received approval from the local ethics commission. This study was approved by Istanbul Medipol University. Approval Number: E-10840098-202.3.02-771, Date: 27 January 2025.

### 2.2. Definitions

The defining characteristics of AF were the absence of P waves and the presence of abnormal RR intervals on a standard 12-lead ECG or 24 h ambulatory ECG lasting more than 30 s. Paroxysmal AF represents a specific type of AF that manifests within 7 days of its initial onset. In contrast, patients experiencing persistent AF endure an arrhythmia that lasts longer than 7 days, which may include episodes that are amenable to termination through cardioversion, whether pharmacological or electrical, beyond this timeframe [7].

HRV analysis evaluates various features categorized into three domains: frequency domain, time domain, and nonlinear methods. Time-domain parameters of HRV indicate the intervals between successive heartbeats. Key time domain parameters include the standard deviation of normal-to-normal intervals (SDNN), which reflects the overall variability in the RR interval series; the root mean square of successive differences (RMSSD); and the percentage of adjacent RR intervals differing by more than 50 milliseconds (msec) (pNN50), which signals parasympathetic modulation of the heart. Additionally, the standard deviation of average normal-to-normal (NN) intervals for each 5 min segment throughout a 24 h recording (SDANN) is calculated and presented in msec, similar to the SDNN. The SDNN index represents the average of the standard deviations of all NN intervals calculated for each 5 min segment of a 24 h HRV dataset. Frequency domain analysis is a key technique for measuring HRV, as it breaks down signals into frequency bands linked to specific components of the autonomic nervous system. Typically, this analysis focuses on two primary frequency bands: low frequency (LF), ranging from 0.04 to 0.15 Hz, and high frequency (HF), ranging from 0.15 to 0.40 Hz. The HF band indicates parasympathetic modulation, while the LF band is associated with both sympathetic and vagal tone. The LF/HF ratio serves as a useful measure to evaluate sympatho-vagal balance, with a lower LF/HF ratio indicating parasympathetic dominance and a higher ratio suggesting sympathetic dominance. Furthermore, nonlinear methods are thought to offer a deeper understanding of the complex interactions between the parasympathetic and sympathetic branches of HRV. These nonlinear methods include techniques like detrended fluctuation analysis (DFA), power law relationship analysis, approximation entropy, and Point Correlation Dimension (PD2i), which have proven effective in providing predictive insights [21,22].

### 2.3. Statistical Analysis

All statistical analyses were performed using R statistical software, version 4.3.3, developed by the Institute for Statistics and Mathematics in Vienna, Austria. The Kolmogorov–Smirnov test was utilized to determine whether the data followed a normal distribution. Categorical data are presented as counts and percentages. Depending on the situation, either Fisher’s exact test or the chi-square (X^2^) test was used to compare categorical variables between groups. Continuous variables with normal distributions are reported as means (standard deviation, SD), while non-normally distributed data are presented as medians (interquartile range, IQR). The independent Student’s *t*-test and the Mann–Whitney U test were employed to compare continuous variables between groups.

Our objective was to develop a risk prediction nomogram based on 24 h ambulatory ECG parameters. We systematically included all relevant 24 h ambulatory ECG variables in a multivariable logistic regression analysis. To effectively manage overfitting and isolate the most significant predictors of AF development, we implemented the Least Absolute Shrinkage and Selection Operator (LASSO) penalized shrinkage. Through this analysis, we identified four critical variables—HF, LF, SDNN24 h, and PNN50—that were integrated into the multivariable model. We evaluated multicollinearity rigorously, using tolerance values (with a threshold of 0.1) and the variance inflation factor (VIF > 3). The variables in our multivariable model were definitively ranked by their importance based on chi-square (X^2^) values. Following this, we developed a nomogram to estimate the risk of AF development in patients. To ensure the nomogram’s generalizability to new datasets, we conducted an in-depth evaluation using a calibration plot and performed internal validation via bootstrapping with a sample of 300 patients. We assessed its discriminative ability by generating a receiver operating characteristic (ROC) curve and calculating the area under the curve (AUC). Furthermore, a comprehensive decision curve analysis was executed to ascertain the net clinical benefit of the nomogram compared to a strategy of treating all or none of the patients. We established statistical significance with a two-sided *p*-value < 0.05 and a 95% confidence interval (CI).

## 3. Results

A total of 187 patients with moderate to high PVC burdens were included in the final analysis. During a median follow-up period exceeding five years, 30 patients (16.0%) developed new-onset AF, while the remaining 157 patients (84.0%) remained free of AF.

Patients who acquired AF were substantially older than those who did not (median age: 68.0 vs. 58.0 years, *p* < 0.001) and had a higher prevalence of male gender (73.3% vs. 51.6%, *p*: 0.046). HT was substantially more prevalent in the AF group (73.3% vs. 43.9%, *p*: 0.006), but the prevalence of diabetes mellitus, smoking status, coronary artery disease, and chronic obstructive pulmonary disease did not exhibit significant differences between the groups. A history of cerebrovascular accident and prior catheter ablation were significantly more prevalent among patients who acquired AF (16.7% vs. 1.9%, *p*: 0.003 and 16.7% vs. 1.3%, *p*: 0.001, respectively). The echocardiographic evaluation demonstrated substantial structural and functional disparities between the groups. Patients with AF demonstrated a markedly enlarged left atrial anteroposterior (LAAP) diameter (45.0 vs. 37.0 mm, *p* < 0.001), heightened left ventricular end-diastolic dimension (LVEDD) (49.5 vs. 48.0 mm, *p*: 0.049), and augmented interventricular septal (IVS) and posterior wall (PW) thickness (both *p* < 0.001). Moreover, the left ventricular ejection fraction (LVEF) was markedly reduced in the AF group (50.0% compared to 60.0%, *p* < 0.001). Regarding laboratory parameters, patients with AF demonstrated higher serum creatinine levels (0.95 vs. 0.80 mg/dL, *p* = 0.005) and lower hemoglobin (Hb) and blood urea nitrogen (BUN) concentrations (*p*: 0.048 and *p* < 0.001, respectively). Thyroid function tests and glycemic indices did not differ significantly between groups.

Table 1 illustrates that the analysis of 24 h ambulatory Holter ECG data revealed statistically significant increases in several parameters among patients with AF.

Analysis of 24 h ambulatory ECG-derived HRV parameters demonstrated marked differences between groups. Patients who developed AF exhibited significantly higher values of several time-domain HRV indices, including SDNN index (61.5 vs. 53.0 ms, *p*: 0.026), RMSSD (65.5 vs. 30.0 ms, *p*: 0.003), and pNN50 (23.0 vs. 8.0, *p*: 0.002). Similarly, frequency-domain analysis revealed significantly higher total power (TP) (3496 vs. 2385 ms^2^, *p*: 0.020) and LF power (1385 vs. 744 ms^2^, *p*: 0.010) among patients who developed AF. Although HF power was numerically higher in the AF group, this difference did not reach statistical significance (*p*: 0.114). No significant differences were observed in SDNN over 24 h or SDANN index. Table 1 provides a comprehensive comparison of the demographic and clinical characteristics of the patients.

**Figure 1:**
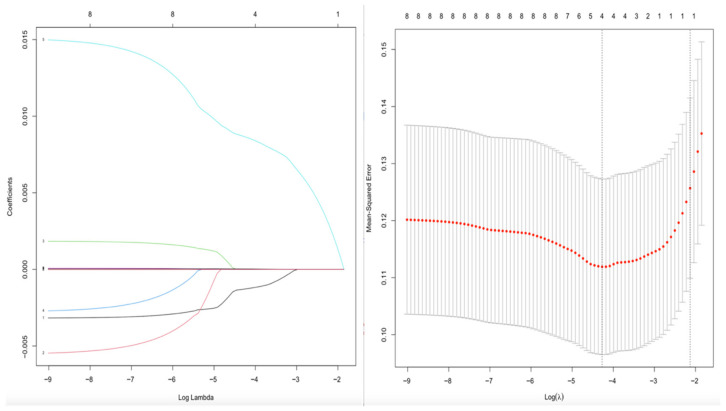
Penalized Least Absolute Shrinkage and Selection Operator (LASSO) regression for variable selection. In the (**left**) panel, each colored line represents the coefficient path of an individual predictor as a function of log λ. In the (**right**) panel, red dots indicate the cross-validated error with ±1 standard error (gray bars). Vertical dotted lines denote the optimal penalty parameters (λ min and λ_1se).

**Figure 2:**
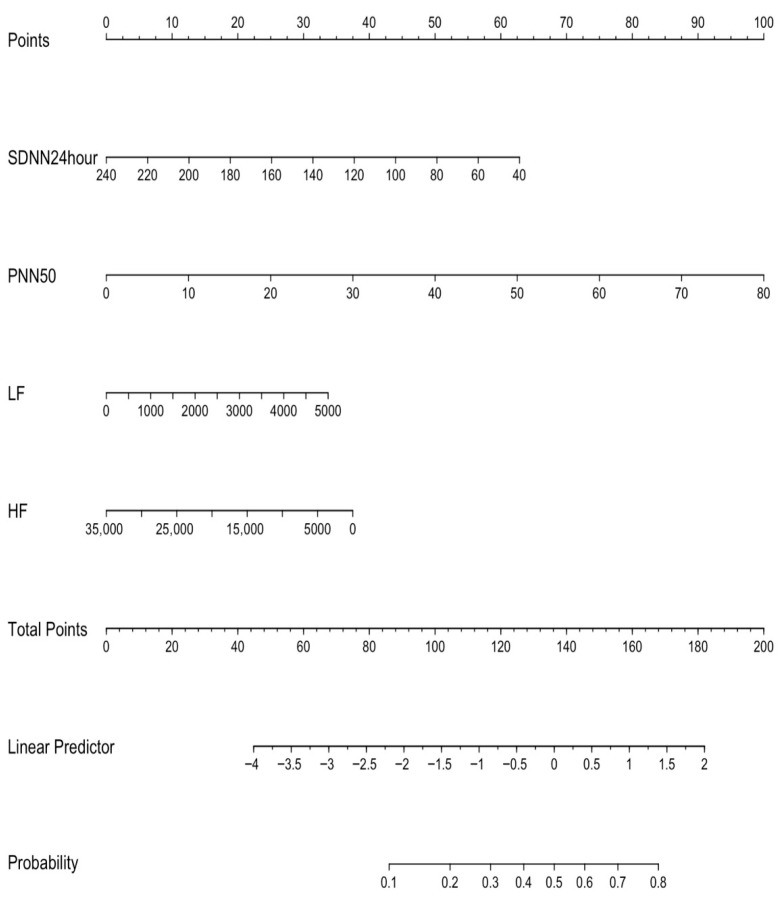
A risk prediction nomogram. Abbreviations: SDNN, standard deviation of normal-to-normal intervals (SDNN); PNN50, the proportion of adjacent RR intervals differing by more than 50 ms; LF, low frequency; HF, high frequency.

**Figure 3:**
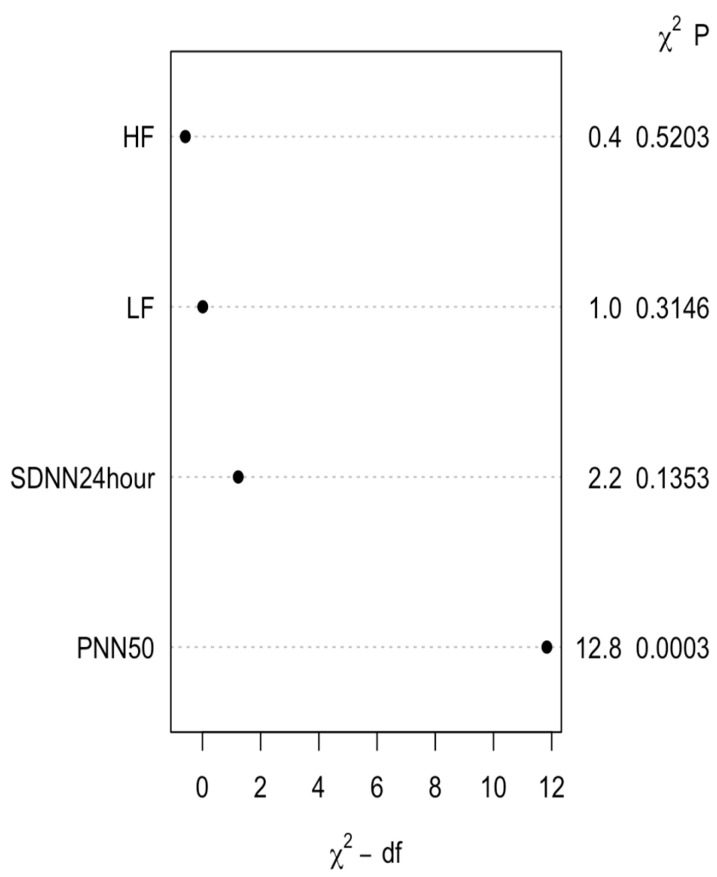
Multivariable model based on chi-square (X^2^) values. Abbreviations: SDNN, standard deviation of normal-to-normal intervals (SDNN); PNN50, the proportion of adjacent RR intervals differing by more than 50 ms; LF, low frequency; HF, high frequency.

To avoid overfitting and multicollinearity, and for variable selection analysis, a penalized LASSO regression with 10-fold cross-validation was applied. Variables with non-zero coefficients at the optimal λ were entered into the final multivariable logistic regression model, as shown in Figure 1. A risk prediction nomogram was created based on four variables, namely HF, LF, SDNN over 24 h, and PNN50, to estimate the risk of AF in PVC patients (Figure 2). Among these variables, PNN50 emerged as the strongest predictor in the multivariable model, as indicated by chi-square (X^2^) values (Figure 3).

**Figure 4:**
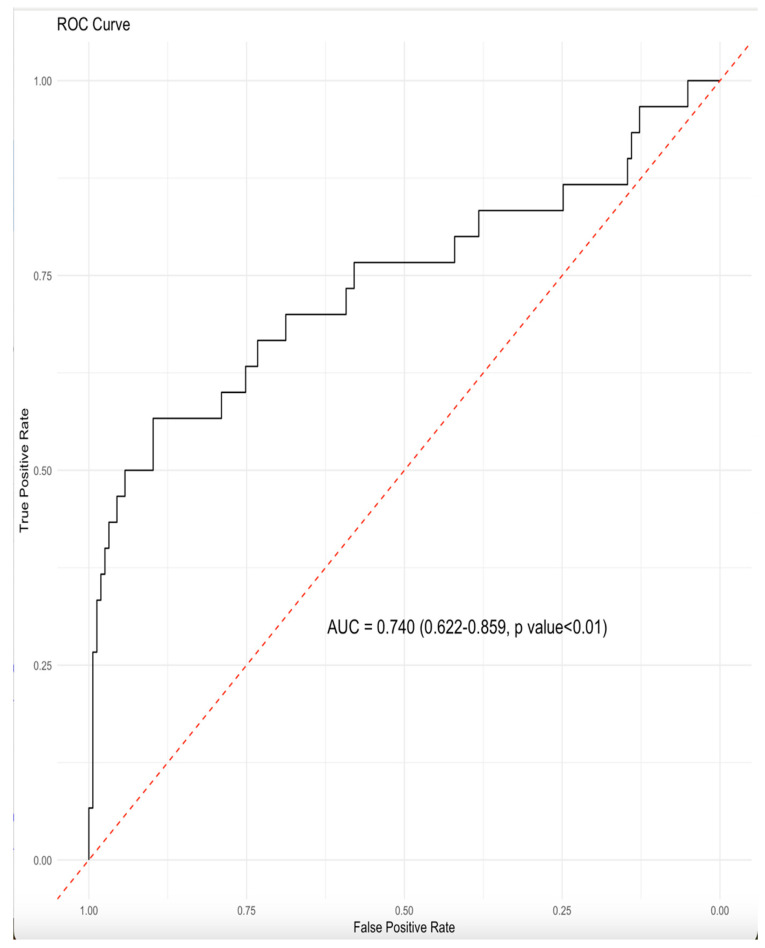
A receiver operating characteristic (ROC) curve analysis. Abbreviations: AUC, area under the curve; CI, confidence interval.

**Figure 5:**
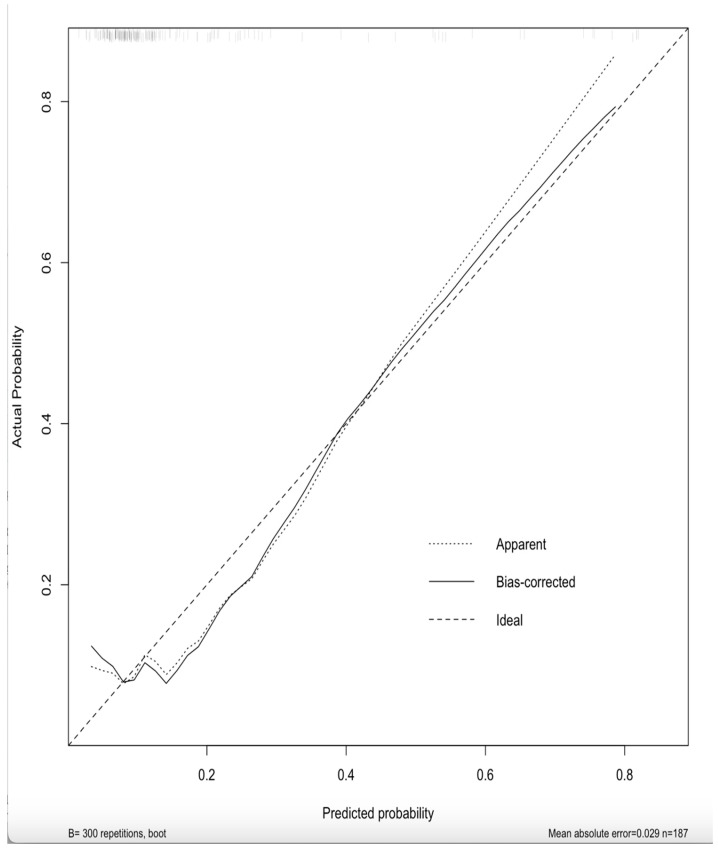
Figure for generalization of calculation: the calibration plot.

**Figure 6:**
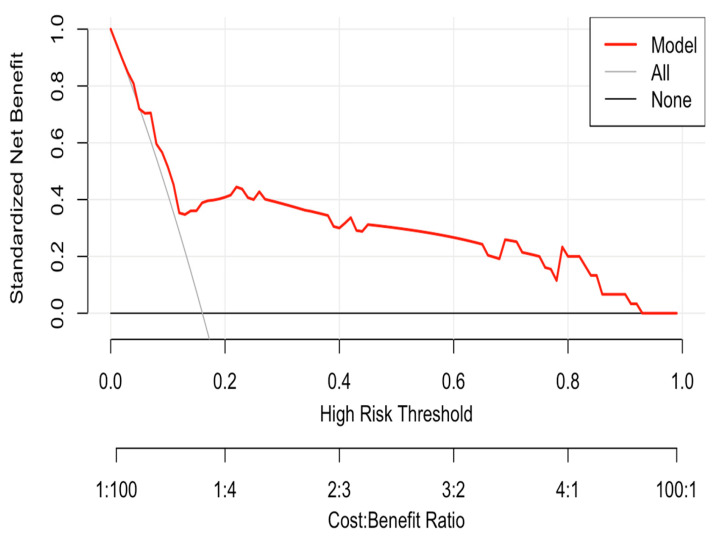
Decision curve analysis.

The ROC curve analysis demonstrated that the nomogram had discriminatory power in distinguishing AF patients from non-AF patients (Figure 4). The calibration plot confirmed that the nomogram generalizes the data (Figure 5). Finally, decision curve analysis indicated that the nomogram provides a net clinical benefit for detecting AF in patients when the baseline threshold risk exceeds 15% (Figure 6).

The apparent C-index was determined to be 0.74. Internal validation was performed using bootstrap resampling with 300 iterations. The optimism-corrected discrimination (optimism-corrected C-index ≈ 0.71) indicated limited optimism. An optimism-corrected calibration slope of 0.91 and a calibration intercept of −0.13 were determined. Overall model accuracy assessed by the Brier score was acceptable (corrected B: 0.107)

## 4. Discussion

In our current study of patients with PVCs monitored by 24 h ambulatory ECG, we observed that specific HRV parameters—SDNN index, RMSDD, PNN50, TP, and LF—were significantly increased in individuals who subsequently experienced AF compared to those who did not. These data suggest a potential link between increased autonomic variability and the onset of AF, indicating that autonomic dysfunction plays a significant role in the development of arrhythmias.

Our findings reveal that increased variability in autonomic regulation, especially parasympathetic and overall autonomic modulation, may predispose patients to AF. Although diminished HRV has conventionally been linked to negative cardiovascular outcomes, the correlation between HRV and arrhythmic conditions may vary from other cardiovascular disorders owing to autonomic instability [14,23,24]. PVCs are often triggered by overstimulation of the sympathetic nervous system, and a high frequency of PVCs indicates increased sympathetic activity [25]. A study by Dong Y et al. showed that reduced HRV in time-domain parameters, specifically SDNN, SDANN, RMSSD, and PNN50, can signal the formation of PVCs. These parameters were significantly lower in patients with PVCs compared to those without, indicating heightened sympathetic activity and decreased vagal activity among PVC patients [26]. The association between AF and HRV may vary somewhat from that of PVCs. According to the review of Andresen D et al., the start of AF is influenced by circadian changes in HRV. Vagal-mediated AF is the most prevalent type, usually commencing with bradycardia and occurring more frequently during nocturnal hours. Sympathetic-mediated AF is precipitated by stress, correlated with increased extrasystoles and tachycardia, and generally manifests during daytime hours [27]. Our study results indicated that, similar to vagal-mediated AF, which is more prevalent, the time-domain measures of HRV reflecting parasympathetic modulation of the heart—specifically, the SDNN index, RMSSD, and PNN50—were elevated in the AF group. Likewise, whereas sympathetically mediated AF is less prevalent, LF, a frequency-domain measure signifying sympathetic modulation, was also increased in this cohort. Although seemingly paradoxical, our patient cohort comprised PVC patients exhibiting elevated sympathetic activation, and sympathetically mediated AF is predominantly linked to recurrent extrasystoles and elevated heart rate, as shown in our patient group. A recent study by Grégoire JM et al. identified a notable elevation in both HF, RMSSD, and pNN50, as well as LF prior to the beginning of AF, corroborating our findings [28]. Similarly, the study conducted by Jin H et al. revealed that RMSSD was elevated in patients diagnosed with AF [29]. In another study conducted by Sagnard A et al., patients who experienced AF following acute myocardial infarction exhibited diminished LF/HF ratios, although PNN50 and RMSSD values were markedly elevated [30].

In addition to HRV measurements, we observed structural changes in the hearts of patients who experienced AF. Specifically, there were significant increases in LAAP and LVEDD, as well as thickening of the IVS and PW in this group. Furthermore, the LVEF was notably reduced. These structural changes may suggest underlying atrial remodeling and increased atrial strain, both of which are recognized factors in the development of AF. The enlargement of the LA is considered a predictor of AF, as it is associated with both electrical and structural remodeling, increasing the risk of atrial ectopic beats and re-entry circuits [31,32,33].

Our findings suggest that patients who developed AF tended to be older and primarily male, which aligns with the established epidemiology of AF. Aging is associated with electrophysiological changes, increased fibrosis, and structural remodeling, all of which contribute to a higher risk of AF in older patients. Additionally, male gender has been identified as a risk factor for AF, likely due to differences in atrial size and hormonal influences [34].

Another notable finding in our study is the higher prevalence of previous catheter ablation among patients who later developed AF. This may indicate a subgroup of patients with more advanced arrhythmic disease or those who have previously undergone ablation for other arrhythmias. Such procedures could lead to changes in the atrial substrate that predispose patients to AF [35]. Additionally, we observed lower Hb and BUN levels in the AF group. Lower Hb levels may indicate underlying anemia or chronic disease states, which can contribute to myocardial hypoxia, oxidative stress, and increased sympathetic activation. These factors promote the development of atrial arrhythmias [36]. In patients with AF, serum creatinine levels increased, although BUN levels were lowered. Increased serum creatinine is a recognized risk factor for AF, and our study’s findings align with existing literature [37]. However, the low BUN level seen in this group may appear contradictory. This notable disparity may stem from the greater influence of non-renal factors on BUN compared to creatinine. BUN is substantially affected by protein metabolism, muscle mass, hepatic urea production, hydration state, nutrition, and neurohormonal activation, whereas creatinine predominantly reflects glomerular filtration and chronic renal microvascular injury. The markedly elevated prevalence of elderly, low-ejection fraction, and hypertensive patients in the AF group may have contributed to diminished protein consumption, reduced muscle mass, and fluctuating hydration levels, resulting in decreased BUN levels within this patient population [38,39,40,41].

The findings collectively suggest that measures of HRV, structural changes in the heart, demographic factors, and biochemical markers may serve as potential predictors of AF risk in patients with PVCs. This nomogram may assist in risk stratification and hypothesis generation, pending external validation. Elevated HRV observed in the AF group may indicate increased autonomic instability, which could contribute to the onset and persistence of AF episodes. Future prospective studies with larger cohorts are essential to further investigate the molecular mechanisms linking HRV changes and structural heart alterations to the onset of AF in this patient group. Prompt diagnosis of high-risk patients may facilitate the implementation of strategies to prevent the development of AF and its associated consequences. Additionally, future studies should explore these relationships and evaluate whether targeted treatments, such as autonomic modulation medications, careful management of risk factors, or improved post-ablation monitoring, might reduce the risk of developing AF in patients who frequently experience PVCs.

### Limitations

It is crucial to recognize several limitations inherent in our analysis, which may affect the interpretation of our findings. This study was conducted as a retrospective study at a single center, which inherently limits the generalizability of the results due to potential selection bias and the lack of diversity in the patient population. The sample size was also limited, reducing the statistical power to detect significant differences or associations. Although the number of AF events was limited, internal validation results indicate adequate model stability; nevertheless, external validation remains necessary. We acknowledge the absence of external validation as a limitation of this study. Furthermore, the HRV parameters we analyzed, particularly the HF and LF components, can be significantly influenced by respiratory rate and depth. Unfortunately, we did not measure or standardize the respiratory characteristics of the study participants, which could lead to variability in the HRV data. This lack of control could confound our results and limit the clarity of any associations drawn between HRV and AF risk. Additionally, our study participants received ECG and 24 h ambulatory ECG monitoring; however, they were not subjected to continuous rhythm monitoring, such as an implanted loop recorder, throughout follow-up. This limitation raises the possibility that some cases of AF may have been missed, potentially skewing the prevalence rates reported in our findings. Given these considerations, we consider that future research should be prospective in nature, involving multiple centers to ensure a more equitable distribution of diverse patient populations. Larger cohort sizes, coupled with extended follow-up periods, will be essential to validate our findings and further elucidate the relationships between HRV and AF onset.

## 5. Conclusions

Our study revealed that the SDNN index, RMSDD, PNN50, TP, and LF of HRV parameters were higher in patients with AF who also had PVCs monitored using a 24 h ambulatory ECG. Predicting the onset of AF in patients with PVCs using HRV measurements could provide an opportunity to prevent the serious complications associated with AF. Therefore, HRV should not be ignored in patients with PVCs, and the relevant parameters for those at risk of AF should be carefully evaluated.

## Figures and Tables

**Table 1:** Comparison of demographic and clinical features of patients based on the development of atrial fibrillation.

	AF Absent	AF Present	*p* Value
	*n*: 157	*n*: 30	
Age, y	58.0 [48.0; 66.0]	68.0 [58.5; 79.0]	<0.001
Male gender	81 (51.6%)	22 (73.3%)	0.046
Hypertension	69 (43.9%)	22 (73.3%)	0.006
Diabetes mellitus	26 (16.6%)	8 (26.7%)	0.291
Smoking	27 (17.2%)	8 (26.7%)	0.336
Coronary artery disease	40 (25.5%)	10 (33.3%)	0.506
Chronic obstructive pulmonary disease	7 (4.46%)	1 (3.33%)	1.000
Ejection fraction, %	60.0 [55.0; 60.0]	50.0 [40.0; 55.0]	<0.001
LAAP, mm	37.0 [32.0; 42.0]	45.0 [40.2; 49.8]	<0.001
LVEDD, mm	48.0 [44.0; 52.0]	49.5 [46.2; 57.0]	0.049
LVESD, mm	31.0 [27.0; 38.0]	33.5 [30.0; 42.8]	0.142
IVS	10.0 [9.00; 11.0]	11.0 [10.0; 13.0]	<0.001
PW	10.0 [9.00; 10.0]	11.0 [10.0; 12.0]	<0.001
Hb (g/dL)	13.6 [12.3; 14.9]	13.0 [11.8; 13.9]	0.048
TSH	1.50 [1.00; 2.40]	1.70 [0.62; 2.72]	0.919
Creatinine (mg/dL)	0.80 [0.70; 0.90]	0.95 [0.78; 1.20]	0.005
Urea (mg/dL)	28.0 [19.0; 36.0]	17.0 [12.2; 20.8]	<0.001
Glucose (mg/dL)	102 [93.0; 113]	110 [95.2; 117]	0.143
Hemoglobin A1C	5.80 [5.50; 6.40]	5.95 [5.50; 6.10]	0.816
PVC, *n*	8041 [min: 3900; max: 14,942]	6306 [min: 2275; max: 16,394]	0.259
Cerebrovascular accident	3 (1.91%)	5 (16.7%)	0.003
Previous ablation	2 (1.27%)	5 (16.7%)	0.001
Mean heart rate	71.0 [64.0; 77.0]	70.5 [58.2; 79.8]	0.684
Min heart rate	47.0 [44.0; 52.0]	46.5 [41.2; 51.0]	0.275
Max heart rate	119 [103; 132]	119 [95.2; 139]	0.837
SDNN 24 h	123 [103; 141]	120 [99.5; 147]	0.375
SDANN index	110 [91.0; 128]	103 [83.8; 118]	0.205
SDNN index	53.0 [42.0; 63.0]	61.5 [46.2; 86.0]	0.026
RMSSD	30.0 [23.0; 44.0]	65.5 [28.8; 95.2]	0.003
PNN50	8.00 [3.00; 17.0]	23.0 [6.75; 57.8]	0.002
TP	2385 [1557; 3631]	3496 [2058; 5779]	0.020
LF	744 [383; 1150]	1385 [458; 2652]	0.010
HF	5352 [3344; 8446]	6947 [5675; 9153]	0.114

Abbreviations: AF, atrial fibrillation; LAAP, left atrium anteroposterior; LVEDD, left ventricular end-diastolic dimension; LVESD, left ventricular end-systolic dimension; IVS, interventricular septum; PW, posterior wall; Hb, hemoglobin; TSH, thyroid-stimulating hormone; PVC, premature ventricular complex; Min, minimum; Max, maximum; SDNN, standard deviation of normal-to-normal intervals (SDNN); RMSDD, root mean square of successive differences; PNN50, the proportion of adjacent RR intervals differing by more than 50 msec; TP, total power; LF, low frequency; HF, high frequency.

## Data Availability

The datasets used in this study are not openly accessible; however, the relevant author may make them available upon request.
